# Ocular scedosporiosis: A case series

**DOI:** 10.1016/j.ajoc.2024.102190

**Published:** 2024-10-16

**Authors:** Marcus L. Turner, Minh Nguyen, Julie Schallhorn, Gerami D. Seitzman

**Affiliations:** a Department of Ophthalmology, University of Washington, Seattle, WA, USA; b Department of Ophthalmology, University of California San Francisco, San Francisco, CA, USA; c Proctor Foundation, University of California San Francisco, San Francisco, CA, USA

**Keywords:** Scedosporium, Infectious keratitis, Infectious scleritis

## Abstract

**Purpose:**

To report five cases of ocular scedosporiosis with associated predisposing factors, treatment courses, and clinical outcomes.

**Observation:**

This case series consists of 5 patients diagnosed with ocular scedosporiosis. Two patients were female and 3 were male. The average age was 68.4 years (range 53–85). Four of the 5 had a clear history of ocular surgery or ocular trauma with organic foreign material. Two developed sclero-keratitis. Two had cornea-only involvement. There was 1 scleritis-only case. Patients with scleritis required topical and systemic treatment. Patients with only keratitis were treated topically. The two patients with sclero-keratitis ultimately progressed to eye removal despite maximal therapy.

**Conclusion and importance:**

Ocular scedosporiosis is exceedingly rare, especially in the Unites States. We highlight similarities and differences of five ocular scedosporiosis cases. Most cases involved either ocular surgery or contamination with ground or plant matter. This case series highlights the challenge of the diagnosis and the aggressivity of this disease.

## 1 Introduction

*Scedosporium* is an emerging infectious disease in both immunocompetent and immunocompromised patients.^1^ *Scedosporium* spp. are ubiquitous filamentous fungi found in soil and polluted water. There are two primary clinically relevant species: *S. apiospermum and S. prolificans*. Both cause a wide range of local and systemic diseases.^1^ Ocular scedosporiosis is particularly rare.^2^^,^^3^ Keratitis is the most reported ocular manifestation of *S. apiospermum*.^4^ Treatment response to ocular scedosporiosis exhibits high variability.^5^ This case series describes five cases of ocular scedosporiosis. All cases were diagnosed and treated at the University of California at San Francisco (UCSF) between 2014 and 2022.

## 2 Findings

### 2.1 Case 1

A 71-year-old woman presented with a 3-month history of slowly worsening left eye redness and irritation. Past medical history was significant for hypercholesterolemia. Past ocular history was significant for left eye pterygium removal with mitomycin C (MMC) and amniotic membrane graft 4 years prior to presentation. Three months prior to presentation, dirt from a bag of potting soil blew into her left eye. This caused intermittent ocular irritation. After 2 months, she was evaluated by a local ophthalmologist. Initially diagnosed with iritis, she was treated with topical prednisolone acetate 1 %. The left eye pain and redness worsened, and she was diagnosed with scleritis. Topical moxifloxacin 0.5 % and oral indomethacin were started. The scleritis worsened and the patient was referred. On presentation, her left eye best-corrected visual acuity (BCVA) was 20/40. Intraocular pressure (IOP) was 25 mmHg. Slit lamp biomicroscopy of the left eye revealed a focal area of avascular and necrotic nasal sclera with an overlying calcific plaque. The adjacent nasal cornea demonstrated a stromal infiltrate with marked thinning. Numerous fungal filaments were seen on Gram stain. Oral voriconazole 200mg twice daily, topical natamycin 5 % every hour and moxifloxacin 0.5 % four times daily were initiated. The disease progressed. Sub-conjunctival and intracameral amphotericin B injection were administered. After the intracameral injection, the area of corneal thinning progressed to a perforation and was temporized with glue. The next day, the patient received a corneal scleral patch graft in the operating room (OR). Cultures from excised tissue grew *Scedosporium apiospermum*. The infection progressed rapidly, now with scleral perforation and uveal prolapse. The patient returned to the OR for a repeat patch graft at which time a choroidal abscess was noted, cultured, and also grew *S. apiospermum*. Given severe pain and rapid dissemination of the fungal infection despite topical, injection, and systemic antifungals, the patient opted for evisceration.

### 2.2 Case 2

**Fig. 1:**
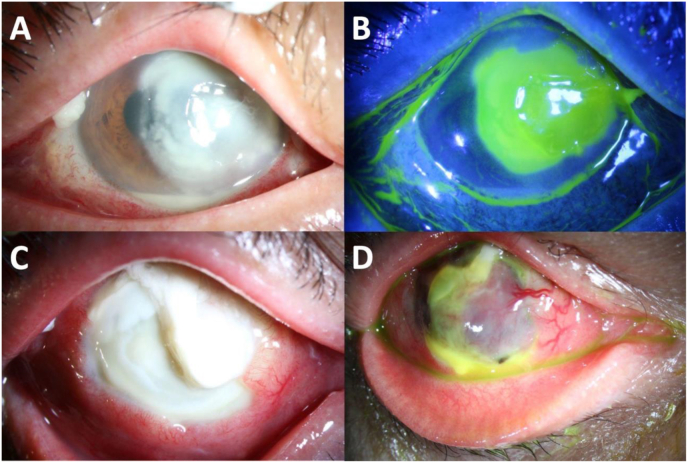
Slit lamp photographs of case 2. A-B. Initial presentation with scleral abscess, large corneal epithelial defect with underlying stromal infiltrate and hypopyon. C. Progression to peripheral corneal thinning and massive stromal necrosis. D. Perforation and uveal prolapse in a blind, painful eye requiring evisceration.

A 71-year-old man presented with a three-week history of right eye pain, blurry vision, and redness. His past medical history was significant for poorly controlled diabetes, hypertension, and hypercholesterolemia. He reported no significant ocular history. Symptoms began soon after a dental extraction. Additionally, the patient is a recreational gardener but cannot recall an isolated incident of eye trauma. Upon onset of symptoms, he was evaluated by a local ophthalmologist and treated for presumed infectious keratitis with topical ofloxacin. Though prescribed hourly, the patient reported taking the antibiotic drops only a few times a day. Symptoms reportedly improved initially. However, after two weeks, the eye pain and redness worsened significantly prompting a referral. On presentation, right eye BCVA was hand motion (HM) and IOP was 10 mmHg. Slit lamp biomicroscopy revealed a large central full thickness corneal stromal infiltrate with overlying epithelial defect and a small nasal scleral abscess. (Fig. 1A–B). The anterior chamber (AC) was robustly inflamed with 4+ cells and a 1mm hypopyon. In-office confocal showed no evidence of hyphae or acanthamoeba cysts and immediate gram stain and microbiology cultures were negative. Given the negative confocal and gram stain, moxifloxacin 0.5 % hourly, oral doxycycline 100mg 2 times daily, ciprofloxacin 500mg twice daily, and prednisolone acetate 1 % twice daily were started. Multiple subconjunctival moxifloxacin injections 0.5 % in the area of the scleral abscess were administered. On day 4 after referral-center presentation, cultures grew *Scedosporium apiospermum*. The topical steroid, of which the patient had taken only two drops, was immediately discontinued. The patient was started on oral voriconazole and 1.25 % betadine as a transitional treatment for 3 days until topical natamycin 5 % was available. This stabilized the patient until 1 month later he presented with worsening pain and headache. The scleral abscesses were larger, more numerous, and were drained surgically. Topical voriconazole 1 % every 2 hours was added in addition to oral voriconazole 300mg twice daily. Despite maximal treatment and continued scleral drainage, there was worsening pain, hypopyon, scleral abscess, peripheral corneal thinning, and stromal necrosis (Fig. 1C). Given lack of improvement and examination demonstrating scleral perforation with uveal prolapse (Fig. 1D), the patient opted for evisceration.

### 2.3 Case 3

**Fig. 2:**
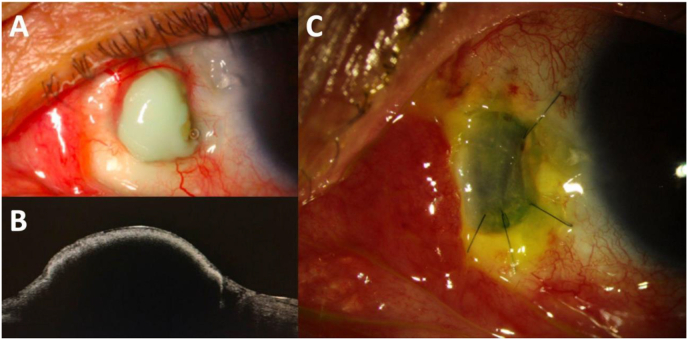
Slit lamp photographs and anterior segment OCT imaging of case 3. A. Large nasal scleral lesion. B. Anterior segment optical coherence tomography of the scleral abscess. C. Corneal patch graft after debridement of the scleral abscess.

An 85-year-old man presented with a 3-week history of worsening left eye pain and discharge. His past medical history was significant for hypertension, hypercholesterolemia, and arthritis. His ocular history was significant for pterygium surgery with irradiation 20 years prior. 10 years after this surgery, a scleral melt around the area of prior pterygium removal necessitated a scleral patch graft. A local ophthalmologist evaluation was concerning for recurrent melt and secondary infection of the scleral patch graft. The patient was referred. On presentation, left eye BCVA was 20/50 and IOP was 10 mmHg. Slit lamp biomicroscopy revealed a nasal scleral lesion (Fig. 2A). The AC was deep and quiet. Anterior optical coherence tomography demonstrated marked elevation of the mass with shadowing of underlying details indicative of significant density of the overlying tissue. (Fig. 2B). After 4 days, cultures from this lesion returned positive for *Scedosporium apiospermum.* The following day he was brought to the OR for emergent scleral debridement with patch graft (Fig. 2C). Oral voriconazole 200mg 2 times daily, topical voriconazole 1 % every 2 hours, moxifloxacin 0.5 % 4 times daily, and natamycin 5 % 4 times daily were started. Pain resolved 1 month post-operatively. The infection resolved and patient maintained a BCVA in the affected eye of 20/25.

### 2.4 Case 4

**Fig. 3:**
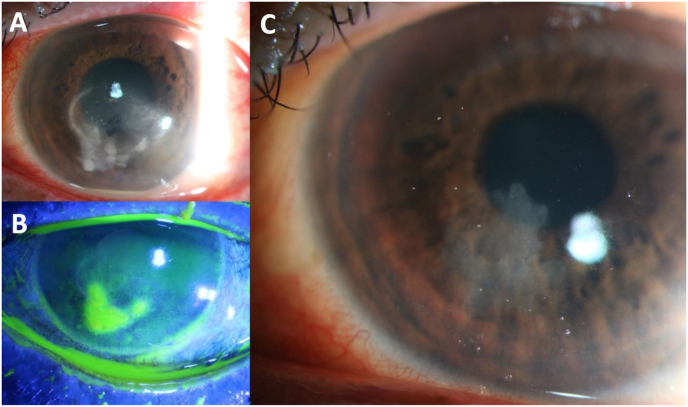
Slit lamp imaging of case 4. A. Keratitis on presentation with multifocal stromal infiltrates and hypopyon. B. 3 × 5mm inferior corneal epithelial defect. C. The remaining scar after corneal ulcer resolved.

A 53-year-old man presented with a 3-day history of left eye irritation, foreign body sensation, and vision loss. He was moving boxes for work and reported that dirt debris flew into his eye. He had no significant medical or ocular history. He presented to a county hospital emergency department and was diagnosed with a corneal ulcer. Moxifloxacin 0.5 % every 2 hours was started, and the patient was referred for evaluation and treatment. On presentation, left eye BCVA was 20/250 and IOP was 14 mmHg. Slit lamp biomicroscopy revealed a 3 × 5mm corneal epithelial defect overlying a large area of patchy central anterior stromal infiltrates, diffuse stromal edema, and an endothelial plaque. A half millimeter hypopyon was present (Fig. 3A–B). Two days later, cultures returned positive for *Scedosporium apiospermum*. Natamycin 5 % every hour was initiated and moxifloxacin 0.5 % was reduced to 4 times daily. Within 5 days, there was significant improvement of the anterior stromal infiltrates, corneal stromal edema, and the endothelial plaque had resolved. By 3 months, the epithelial defect was healed, the inflammation had resolved, and BCVA in the affected eye returned to 20/25 OS (Fig. 3C).

### 2.5 Case 5

**Fig. 4:**
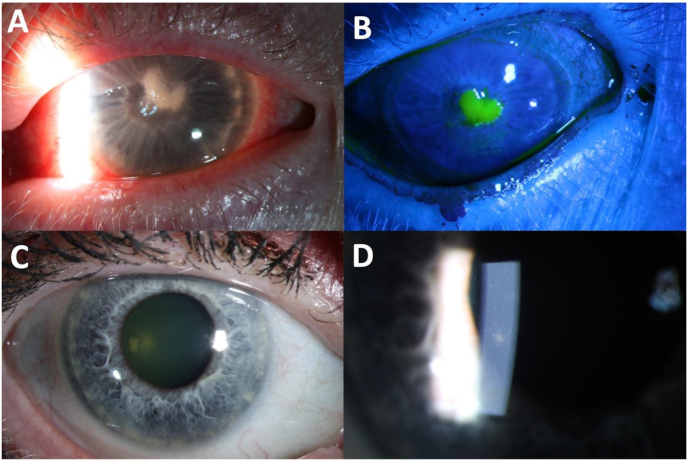
Slit lamp imaging of case 5. A. Keratitis on presentation with stromal infiltrate. B. 2.5 × 3.5mm corneal epithelial defect. C. Resolution of corneal ulcer. D. Faint stromal scar.

A 62-year-old woman presented with three weeks of left eye pain and vision loss. Her past medical history was significant for hypertension. Her ocular history was significant for monovision soft contact lens wear in the unaffected eye only. The patient is a florist and reported getting flower arrangement debris into her eye which caused irritation and a significant amount of subsequent eye rubbing. Locally diagnosed with a corneal ulcer, topical moxifloxacin 0.5 % and tobramycin 0.3 % were initiated hourly. The corneal ulcer worsened, hourly fortified vancomycin 2.5 % and cefazolin 5 % were started, and the patient was referred. On presentation, BCVA was HM and IOP was 6 mmHg. Slit lamp biomicroscopy revealed a central, dense, thick infiltrate with a 2.5 × 3.5mm overlying epithelial defect and diffuse corneal edema (Fig. 4A–B). She was started on topical natamycin 5 % hourly and continued moxifloxacin 0.5 % 4 times daily. Four days later, cultures returned positive for *Scedosporium apiospermum*. The infection responded well to topical natamycin. Within 20 days, the epithelial defect closed. After 2 months the corneal ulcer had healed leaving a central scar. Nuclear sclerosis was present. The patient now wears a scleral lens on the left eye with a BCVA of 20/60 (Fig. 4C–D).

## 3 Discussion

Ocular scedosporiosis is rare and very few case series exist. The largest case series on *Scedosporium* keratitis was conducted in India and reported out of 1792 culture positive fungal keratitis cases, only 10 (0.6 %) were due to *Scedosporium*.^3^ Of these cases, 8 had a history of ocular trauma. Similarly, another study, also from southern India, reported out of 1621 culture-positive fungal keratitis cases, *Scedosporium* was identified in only 13 (0.8 %).^2^

The most common predisposing factors for ocular scedosporiosis include ocular trauma with plant matter or dirt, or prior pterygium surgery.^2^^,^^6^ *Scedosporium* keratitis after posterior subtenon triamcinolone acetonide injection has been reported.^7^ In this series, all patients were immunocompetent, though the one patient (gardener without a clear history of ocular trauma) did have diabetes mellitus which potentially could have rendered him immunocompromised and possibly neurotrophic. It is conceivable this patient could have experienced vegetable matter trauma while gardening but did not have pain because of diabetic neurotrophic corneal changes. Two of five patients had a remote history of pterygium surgery including one with intraoperative irradiation and one with MMC. Both of these cases had infectious scleritis. 3 patients reported plant and/or dirt exposure.

Culture positivity in all 5 cases yielded *S. apiospermum* allowing for targeted antifungal treatments. This highlights the importance of microbiologic investigation in atypical infections such as infectious scleritis and in cases where plant matter injury has been suspected. *In vivo* confocal microscopy can be useful for the diagnosis of filamentous fungal keratitis, though this tool is not universally available and is operator dependent.

Topical natamycin is commonly the first line treatment for keratitis due to *Scedosporium*, as well as other filamentous fungi.^8^ One case series demonstrated healing with topical natamycin monotherapy in 4 out of 5 cases of *Scedosporium* keratitis, and a requirement of combination therapy consisting of natamycin and voriconazole in 3 cases (with 1 case progressing to corneal perforation and penetrating keratoplasty).^3^ All patients in our case series were treated with topical natamycin. With scleral involvement, abscesses were drained and areas of necrotic scleral tissue were deeply debrided. Advanced scleral involvement as a poor prognostic indicator has also been seen in another case series.^2^ Oral voriconazole was used in the 3 of 5 cases with infectious scleritis. Two of those cases had prior pterygium removal and one had a history of poorly controlled diabetes. Despite maximal treatment, 2 cases ultimately progressed to perforation and required evisceration. A similar study from Australia found escalation to systemic voriconazole or ketoconazole was necessary for 6 out of 8 cases with scleral involvement.^6^ In that series, 6 out 7 cases of scleritis had a history of pterygium excision. Pterygium excision, especially when accompanied by a procedure that renders the area avascular such as irradiation or MMC appears to increase risk for infectious scleritis. The remaining 3 eyes in our study had either keratitis or scleritis (not sclero-keratitis). All retained good visual acuity after successful treatment with resolution of infection.

## 4 Conclusions

Fungal keratitis in the United States is uncommon with case numbers varying greatly by geographic location.^9^ Accordingly, fungal keratitis in Northern California is less often detected.^10^, ^11^, ^12^ Furthermore, of all types of filamentous keratitis, ocular involvement with Scedosporium is exceedingly rare, especially in the United States. However, ocular scedosporiosis can progress aggressively and rapidly. Rapid diagnosis is key. Ocular surgery and ocular trauma with organic matter are risk factors. Ocular scedosporiosis can occur in immunocompetent individuals. Combined scleral and corneal involvement, delay in diagnosis, and prior topical steroid treatment can portent a poor prognosis. Even on appropriate antifungal therapy two of five patient in this series progressed to eye removal.

## CRediT authorship contribution statement

**Marcus L. Turner:** Writing – review & editing, Writing – original draft, Visualization, Methodology, Investigation, Formal analysis, Data curation. **Minh Nguyen:** Writing – review & editing, Writing – original draft, Supervision, Methodology, Investigation, Data curation, Conceptualization. **Julie Schallhorn:** Writing – review & editing, Methodology, Investigation, Data curation, Conceptualization. **Gerami D. Seitzman:** Writing – review & editing, Writing – original draft, Supervision, Methodology, Investigation, Formal analysis, Data curation, Conceptualization.

## 5 Patient consent

Consent to publish the case series was not obtained. This series does not contain any personal information that could lead to the identification of the patients.

## Declaration of competing interest

The authors declare that they have no known competing financial interests or personal relationships that could have appeared to influence the work reported in this paper.

The authors have no conflict of interest.
